# Crystal Structure Analysis and the Identification of Distinctive Functional Regions of the Protein Elicitor Mohrip2

**DOI:** 10.3389/fpls.2016.01103

**Published:** 2016-07-26

**Authors:** Mengjie Liu, Liangwei Duan, Meifang Wang, Hongmei Zeng, Xinqi Liu, Dewen Qiu

**Affiliations:** ^1^State Key Laboratory for Biology of Plant Diseases and Insect Pests, Institute of Plant Protection – Chinese Academy of Agricultural SciencesBeijing, China; ^2^State Key Laboratory of Medicinal Chemical Biology, Department of Biochemistry and Molecular Biology, College of Life Sciences, Nankai UniversityTianjin, China

**Keywords:** MoHrip2, crystal structure, truncated mutant, hypersensitive response, disease resistance

## Abstract

The protein elicitor MoHrip2, which was extracted from *Magnaporthe oryzae* as an exocrine protein, triggers the tobacco immune system and enhances blast resistance in rice. However, the detailed mechanisms by which MoHrip2 acts as an elicitor remain unclear. Here, we investigated the structure of MoHrip2 to elucidate its functions based on molecular structure. The three-dimensional structure of MoHrip2 was obtained. Overall, the crystal structure formed a β-barrel structure and showed high similarity to the pathogenesis-related (PR) thaumatin superfamily protein thaumatin-like xylanase inhibitor (TL-XI). To investigate the functional regions responsible for MoHrip2 elicitor activities, the full length and eight truncated proteins were expressed in *Escherichia coli* and were evaluated for elicitor activity in tobacco. Biological function analysis showed that MoHrip2 triggered the defense system against *Botrytis cinerea* in tobacco. Moreover, only MoHrip2M14 and other fragments containing the 14 amino acids residues in the middle region of the protein showed the elicitor activity of inducing a hypersensitive response and resistance related pathways, which were similar to that of full-length MoHrip2. These results revealed that the central 14 amino acid residues were essential for anti-pathogenic activity.

## Introduction

Plants survive under adverse circumstances including various of biotic factors because of a very effective immune system (Bernoux et al., 2011). The plant immune system is complex and consists mostly of two major branches of defense (Jones and Dangl, 2006; Mishra et al., 2012; Hou et al., 2013). One branch involves plant cell surface-located pattern recognition receptors (PRRs) that recognize pathogen-associated molecular patterns (PAMPs) and is named PAMP-triggered immunity (PTI). Another branch involves intracellular plant NLRs [nucleotide-binding, leucine-rich repeat (LRR) receptors] that recognize pathogen-secreted effectors and is called effector-triggered immunity (ETI). Understanding plant immunity mechanisms might provide new approaches to protect agricultural production (Dodds and Rathjen, 2010). The plant immune system is based on the effective detection and recognition of different inducers from the environment (Shamrai, 2014). As main inducers, elicitors (the term is defined by Dodds and Rathjen, 2010) to refer to both PAMPs and effectors in the context of this paper) have been intensely studied in the plant immunology field. In the last few decades, substantial progress has been made in the study of elicitors, including the identification of new elicitors and their corresponding receptors in plants and the analysis of their biological functions *in vitro* or in transgenic plants. Such work has revealed the molecular mechanisms of induced immunity that underlie elicitor recognition, defense-related signaling transduction mechanisms, the defense response network and the evolution of plant immunity, among other processes (Dodds and Rathjen, 2010; Hwang et al., 2012; Hou et al., 2013; Chuang et al., 2014; Shamrai, 2014).

Furthermore, studies examining the crystal structures and functional regions of elicitors and receptors increasingly represent another intensely researched topic (Wirthmueller et al., 2013), and over the past 20 years, many elicitor structures have been resolved. These crystal structure studies provide evidence for the classification of new proteins and confirm the functions of elicitor proteins on a molecular level. For example, the effector AvrPphB is considered a papain-like cysteine protease to catalyze proteolysis based on its highly similar crystal structure (Zhu et al., 2004). Moreover, the structures of effectors and their receptors are currently being elucidated, and some effector–receptor complex structures have recently been reported (Maqbool et al., 2015), providing insight into their interactions and revealing mechanisms of the plant immune response. For example, the crystal structure of an AtCERK1-ECD (the ectodomain of the chitin elicitor receptor kinase 1 from *Arabidopsis*) in complex with a chitin pentamer showed that the interaction is primarily mediated by a lysine motif and three chitin residues; dimerization of AtCERK1-ECD is induced by chitin and is critical for activating the plant immune system (Liu et al., 2012). Furthermore, structural studies can also be insightful to investigate evolutionary processes. Some inhibitors that act at the plant–pathogen interface evolved from enzymes (Misas-Villamil and Ra, 2008). For example, the *Triticum aestivum* xylanase inhibitor (TAXI) is considered to have evolved from a pepsin-like aspartic protease ancestor based on an analysis of its crystal structure (Sansen et al., 2004). In addition to the ability to trigger plant immunity, elicitors might have other functions, such as the ability to suppress PTI. The identification of functional regions helps elucidate different elicitor functions. Structure–function experiments indicated that the 75 amino acid C-terminal half of AVR3a lacking the RXLR motif was sufficient for avirulence and suppression functions, and other regions showed no effector activity (Bos et al., 2006). Therefore, structure–function relationship studies provide a theoretical foundation to understand in detail the functions of elicitors in the plant immune system and the mechanisms through which they act.

*Magnaporthe oryzae*, a fungal pathogen of rice blast, is the most devastating rice pathogen and causes a destructive decrease in rice production in epidemic years (Dean et al., 2005). To understand the molecular basis of the rice-*M. oryzae* interaction, several elicitors of the fungal pathogen have been identified, including sphingolipid components of the membranes (Koga et al., 1998) and some avirulence (Avr) effectors (Liu et al., 2010). MoHrip2, a novel elicitor isolated from *M. oryzae*, is an exocrine protein. The 459 bp MoHrip2 open reading frame (GenBank accession no. JQ815555.1) encodes a 152 residue polypeptide with an 18 residue signal peptide. In our previous work, we showed that MoHrip2 causes necrotic lesions and triggers early signaling events in the tobacco leaf defense system, and we showed that MoHrip2 also enhanced resistance to *M. oryzae* in rice (Chen et al., 2014). Although, the MoHrip2 amino acid sequence showed up to 70% similarity to several specific plant fungal pathogen proteins (Chen et al., 2014), the specific function of both MoHrip2 and proteins with similar sequences are unknown.

In this study, we solved the crystal structure of MoHrip2 by X-ray crystallography using the single-wavelength anomalous dispersion (SAD) method. Based on the structure, the distinctive functional regions responsible for the hypersensitive response (HS) and the disease resistance of MoHrip2 were narrowed to a short sequence. Our results provide a framework to understand the function of MoHrip2 in the rice-*M. oryzae* interaction and to elucidate the mechanisms that trigger plant immunity. Furthermore, our results have potential as a strategy for using *in vitro* approaches or transgenic plants to control disease.

## Materials and Methods

### Crystallization and Structure Determination of MoHrip2

Native or selenomethionine-labeled MoHrip2 was recombinantly expressed, purified, and crystallized. Protein expression, purification, crystallization, data collection, and structural determination were performed as described previously (Liu et al., 2013). The positions of all four selenium atoms in an asymmetric unit were successfully located from the peak data set, and the preliminary model was readily built following single-wavelength anomalous diffraction (SAD; Dauter et al., 2002) phasing using the Phenix package (Adams et al., 2010). The Phenix refinement program (phenix.refine; Afonine et al., 2012) was used iteratively for refinement. Simulated annealing, positional refinement and B-factor refinement were applied for multiple rounds. The electron density of loop regions became visible gradually as refinement proceeded. Structure validation was performed periodically during refinement by Procheck (Laskowski et al., 1993). Ordered water molecules were added to the structure in the last round of refinement.

### Plasmid Construction and Truncated Protein Expression and Purification

To express truncated mutants of the MoHrip2 protein, DNA sequences encoding different fragments were amplified by PCR from plasmid pMDR18-T-*mohrip2* (Liu et al., 2013) using the primers shown in **Supplementary Table S1**. The PCR cycles included pre-denaturation at 94°C for 5 min; 30 cycles of 94°C for 30 s, 55°C for 30 s, and 72°C for 30 s; and a final extension step at 72°C for 10 min. The reaction product was separated on a 1.0% agarose gel and analyzed with a UV transilluminator (Bio-Rad Laboratories, Hercules, CA, USA). Gel extraction was performed using the EasyPure Quick Gel Extraction Kit (Transgen, Beijing, China) according to the manufacturer’s instructions. Products of different sizes were cloned into the pET-30 TEV/LIC vector (Liu et al., 2013) downstream of a His_6_ tag or into the BamHI/XhoI site of the pET-M3C vector [reconstructed from the pET-32a(+) vector (Novagen) by the Xinqi Liu Laboratory to delete a thrombin recognition site, an S-tag and an enterokinase recognition site] downstream of a His_6_-Trx (thioredoxin) tag.

Recombinant expression vectors were transformed into *Escherichia coli* Codon Plus competent cells. The cells were cultured in LB medium at 37°C to an OD 600 of 0.6, then were induced with 0.2 mM isopropyl β-D-1-thiogalactopyranoside (IPTG) for 12 h at 16°C. Cell harvesting and the subsequent purification of proteins expressed in a soluble form or in inclusion bodies were performed as described previously (Han et al., 2012; Liu et al., 2013) using a Ni-IMAC matrix column. The empty pET-M3C vector was transformed into competent cells to produce an inactive protein preparation, named His_6_-Trx protein. The His_6_-Trx was expressed and purified using the same method and was used as a negative control. The eluted protein was concentrated and diluted with Tris buffer (50 mM Tris, pH 8.0) before detection using SDS-PAGE. The protein concentration was measured using the Bradford method (Bradford, 1976).

### Hypersensitive Response Induction Assay

*Nicotiana benthamiana* and *Nicotiana tabacum* plants (7–8 weeks–old) were used for the HR assay. Full-length and truncated MoHrip2 proteins and a control were diluted to 5 μM in Tris buffer, and 50 μL was infiltrated into the leaves at locations between the midvein and the edge of the leaf with a needleless syringe. HR symptoms were observed in the infiltrated areas after 24 h according to a previously described method (D’Silva and Heath, 1997).

### Disease Resistance Induction Assay

The fungus *Botrytis cinerea* was cultured on potato dextrose agar (PDA; BD Difco, Sparks, NV, USA) plates for 7–10 days at 25°C with a 12 h photoperiod. *N. benthamiana* (5–6 weeks-old) were used for the disease resistance assay. Full-length and mutant proteins at the same concentration mentioned above were infiltrated into one side (separated by the center vein) of leaves using the same method described above. The leaves were cut off of the plants and placed in a 1% agar-medium plate 6 h post-infiltration (hpi). The inoculation experiment was performed according to a previously described method (Chagué et al., 2006). A 5 mm diameter mycelium cube cut from the edge of *B. cinerea* was placed on the other side of the protein-treated leaves between the midvein and the edge of the leaf. The plates were placed in an illumination incubator under the same conditions as the plants but at a high relative humidity (>90%). The disease level was estimated by measuring the diameters or the major axes of the oval necrotic lesions around the inoculation sites 3 days post-inoculation (dpi; Ferrari et al., 2003).

### RNA Extraction and Quantitative Real-Time PCR

*Nicotiana benthamiana* leaves were treated on one side (separated by the center vein) with the proteins described above using the above-described method. The other side of the treated leaves was harvested 6 hpi for RNA extraction and quantitative real-time PCR (qRT-PCR). Total RNA extracted using TRIzol Reagent (Invitrogen, Carlsbad, CA, USA) was reverse-transcribed into cDNA using TransScript All-in-One First-Strand cDNA Synthesis SuperMix for qPCR (Transgen, Beijing, China) according to the manufacturer’s instructions. qRT-PCR was performed using TransStart Green qPCR SuperMix (Transgen, Beijing, China) and an iQ5 real-time PCR instrument (Bio-Rad Laboratories, Hercules, CA, USA). The conditions for qRT-PCR included an initial denaturation at 95°C for 30 s that was followed by 42 cycles of 94°C for 5 s and 60°C for 30 s. Actin was used as the reference gene, and all of the primers (listed in **Supplementary Table S1**) were designed using Beacon Designer 8. qRT-PCR was performed according to the Minimum Information for Publication of qRT-PCR Experiments (MIQE) standard (Bustin et al., 2009).

## Results

### The Crystal Structure of MoHrip2 Reveals a Thaumatin Domain-Like Fold

**FIGURE 1:**
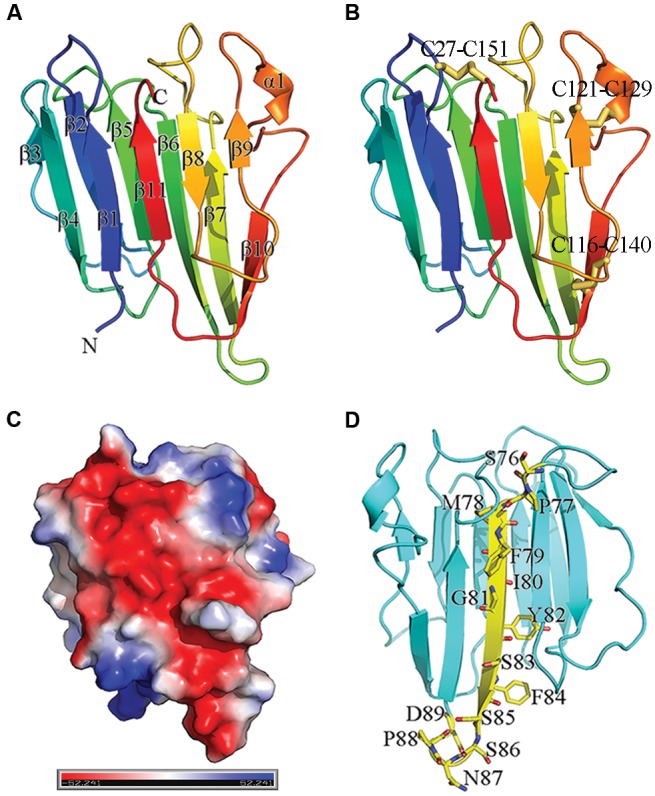
**Structural properties of the MoHrip2 protein.** **(A)** Crystal structure of MoHrip2. There were two identical protein molecules in an asymmetric unit, and only one monomer is shown. MoHrip2 is depicted in ribbon representation (rainbow from blue to red), with the N and C termini and secondary structures labeled. **(B)** Three intramolecular disulfide bridges were formed by the six cysteines indicated in the structure. The disulfide bridges are shown in stick representation. **(C)** Electrostatic potential distribution on the molecular surface of MoHrip2. Blue and red represent negative and positive potentials, respectively. **(D)** A stretch of 14 amino acid residues. The 14 amino residues are shown in stick representation (yellow), with oxygen atoms colored red, and nitrogen atoms colored blue, in the context of the overall structure (cyan ribbon). All of the residues are labeled.

To study the molecular functions of MoHrip2, the full-length protein lacking the signal peptide sequence was expressed, purified and crystallized, and the crystal structure was solved and refined to a resolution of 1.8 Å (PDB 5FID). Refinement statistics are summarized in **Table 1**. The overall architecture is a β-barrel consisting of 11 β-sheets and a very short α-helix (**Figure 1A**). Apart from the N-terminal (β1) and C-terminal (β11) sheet, all of the other β-pleated sheets ran antiparallel within each β-pleated sheet. The short helix was located between the β9 and β10 sheet. MoHrip2 contains six cysteines that are involved in three intramolecular disulfide bridges, which occur between cysteines 27 and 151, 116 and 140, and 121 and 129 (**Figure 1B**). The distribution of the electrostatic surface potentials are shown in **Figure 1C**, which are displayed in scale from -52.24 kTV (red) to +52.24 kTV (blue), with a negative-rich zone in the shown side. A DALI search (Holm and Rosenström, 2010) based on similarity to the MoHrip2 3-dimensional structure revealed that it has similarity to thaumatin/THN domain (PF00314 of Pfam^1^, and SM00205 of SMART^2^)-containing proteins with the highest Z score in the screened proteins and root mean square deviation (RMSD) value more than 2 (**Table 2**). Thaumatin-like proteins (TLPs), including thaumatin, osmotin, zeamatin, pathogenesis-related (PR) proteins and alpha-amylase/trypsin inhibitors, are involved in the plant defense and stress response (Wang et al., 2013), and some TLPs have shown inhibition of fungal growth *in vitro* (Vigers et al., 1992). Thus, these proteins are referred to as pathogenesis-related group 5 (PR5).

**Table 1:** X-ray crystallographic data and refinement statistics for selenomethionine-labeled MoHrip2-L78M.

Date set	MoHrip2-L78M
**Data collection**	
Wavelength (Å)	0.9792
Space group	P21
a (Å)	36.25
b (Å)	43.51
c (Å)	74.00
β°	92.88
Resolution (Å)^a^	1.8 (1.86–1.8)
Molecules per ASU^b^	2
No. of observed reflections^a^	93203 (6670)
No. of unique reflections^a^	21184 (1906)
Completeness (%)^a^	96.6 (86.4)
Redundancy^a^	4.4 (3.5)
R_merge_ (%)^a,c^	5.7 (28.8)
Average I/σ (I)^a^	16.8 (2.3)
**Refinement statistics**	
Resolution (Å)	1.807
No. of reflection	21175
Rwork/Rfree (%)^d,e^	16.30/20.00
No. of atoms	
Protein	2050
Water	402
B-factor (Å^2^)	
Protein	15.22
Water	23.73
r.m.s. deviations	
Bond length (Å)	0.007
Bond angle (°)	1.151
**Ramachandran analysis**	
Favored (%)	96.59
Allowed (%)	3.41
Outliers (%)	0


*^a^Values in parentheses are for the highest resolution shell.*

*^b^ASU = asymmetric unit.*

*^c^R_*merge*_ = Σ|I-<I>|/Σ<I>, in which I is the observed intensity, and <I> is the average intensity of multiple observations of symmetry related reflections.*

*^d^R = Σhkl||Fobs|-|Fcalc||/Σhkl|Fobs|.*

*^e^R_*free*_ is calculated from 5% of the reflections excluded from refinement.*

**Table 2:** Results of pairwise superposition of MoHrip2 with representatives of the thaumatin-like protein family using the DaliLite v3 server (http://ekhidna.biocenter.helsinki.fi/dali_server).

Protein	Source	PDB^a^ entry	Z-score^b^	r.m.s.d^c^ (Å)	No. of aligned residues	Identity (%)
TL-XI	*Triticum aestivum*	3G7M	13.1	2.6	123	18
NP24	*Solanum lycopersicum*	2I0W	12.6	2.2	126	21
Osmotin	*Nicotiana tabacum*	1PCV	12.3	2.3	126	22
Zeamatin	*Zea mays*	1DU5	12.1	2.3	126	17
Beta-1,3-Glucanase	*Streptomyces matensis*	3GD9	9.4	3.0	128	9


*^a^Protein Data Bank. ^b^The Z-score is a measure of the quality of the alignment. The higher the Z-score, the more homologous the structures. A Z-score exceeding 2 is significant. ^c^Root mean square deviation.*

**FIGURE 2:**
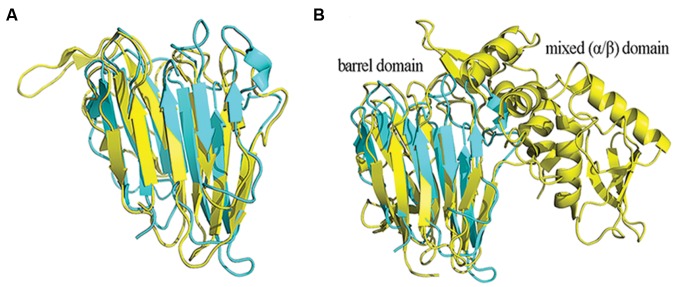
**The comparison of protein crystal structures.** **(A)** Superposition of MoHrip2 with TL-XI (PDB 3G7M) from *Triticum aestivum*. **(B)** Superposition of MoHrip2 with Beta-1,3-Glucanase (PDB 3GD9) from *T. aestivum*. All of the structures are shown in ribbon representation. MoHrip2 is colored cyan, and TL-XI and Beta-1,3-Glucanase are colored yellow.

Thaumatin-like proteins are predicted to consist mainly of β structures, with a high content of β-turns and few helices (Edens et al., 1982). The most similar structure to MoHrip2 was that of a thaumatin-like xylanase inhibitor (TL-XI, PDB 3G7M), and their comparison is shown in **Figure 2A**. TL-XI is a novel type of xylanase inhibitor from wheat (*T. aestivum*) and is a non-competitive inhibitor of glycoside hydrolase family 11 (GH11) xylanase, which shows a unique inhibition specificity (Fierens et al., 2007). The MoHrip2 structure was also similar to that of NP24 (PDB 2IOW; **Supplementary Figure S1A**), which is a salt-induced protein from tomato. NP24 also has β-glucanase activity and antifungal properties and is involved in protecting plant cells from pathogens (Ghosh and Chakrabarti, 2008). The MoHrip2 structure was also similar to two other antifungal proteins, osmotin (PDB 1PCV) and zeamatin (PDB 1DU5; **Supplementary Figures S1B,C**), which were purified from *N. tabacum* and *Zea mays*, respectively. Both of these proteins are involved in fungal plasma membrane permeabilization and are believed to be representatives of a class of plant antifungal proteins (Roberts and Selitrennikoff, 1990; Abad et al., 1996). Other than the thaumatin proteins, the MoHrip2 structure is also similar to the barrel domain of the protein Beta-1,3-Glucanase (PDB 3GD9; **Figure 2B**), which is a GH64 protein from *Streptomyces matensis* (Wu et al., 2009). However, all of the proteins mentioned above show very low primary amino acid sequence homology (range from 9 to 16%) to MoHrip2 (**Supplementary Figure S2**).

### Purified Full-Length and Truncated Forms of MoHrip2 Show Functional Differences in HR Induction

**FIGURE 3:**
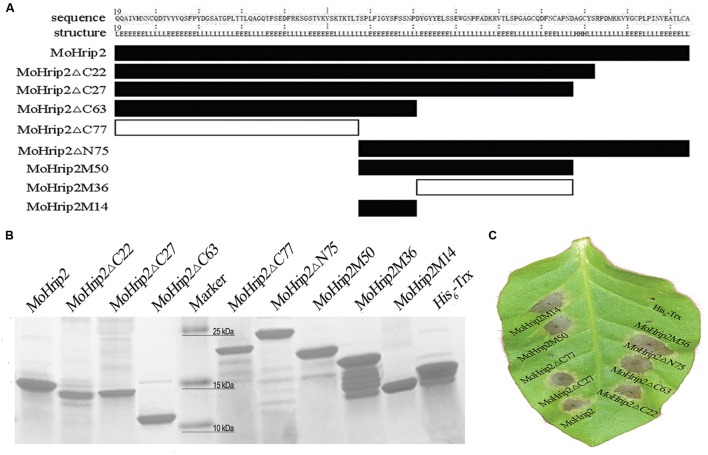
**The purification of truncated MoHrip2 mutants and their ability to induce HR.** **(A)** Diagram of MoHrip2 and the truncated mutants that were used to test different regions of the protein for elicitor activity. The wide strips indicate spans of amino acid resides, and the strip color indicates the HR-inducing ability of different regions. Black indicates HR-inducing ability, and white indicates no HR-inducing ability. **(B)** SDS-PAGE analysis of proteins purified using a Ni-IMAC matrix column. MoHrip2 and the truncated mutants were expressed from different vectors and were coupled to different tags. Proteins 1–4 contained a His_6_ tag. Proteins 5–9 contained a His_6_ tag and a Trx-tag. **(C)** Macroscopic views of the HR symptoms induced by the prepared proteins at a concentration of 5 μM in *Nicotiana tabacum* cv. Samsun NN. The photos were taken 24 h after infiltration.

To determine which region of MoHrip2 is critical for HR induction or for inducing resistance, several truncated mutants were designed using the structurally determined positions of the β-pleated sheet and α-helix (**Figure 3A**). Each of the truncated mutants was expressed, purified and analyzed by SDS-PAGE (**Figure 3B**). The protein with His_6_ tag (MoHrip2, MoHrip2Δ C22, MoHrip2Δ C27, and MoHrip2Δ C63) were expressed in inclusion bodies while the protein with His_6_-Trx (MoHrip2Δ C77, MoHrip2Δ N75, MoHrip2M50, MoHrip2M36, MoHrip2M14, and His_6_-Trx) in a soluble form. The theoretical value of the isoionic point, the number of amino acid residues, the expression vector used, the theoretical molecular weight, and other details of the truncated mutants are described in **Supplementary Table S2**. Initially, amino acid residues were deleted from the C-terminus, and four mutants were generated (MoHrip2Δ C22, MoHrip2Δ C27, MoHrip2Δ C63, and MoHrip2Δ C77). Their abilities to induce HR in tobacco plants were evaluated, and although the first three mutants induced HR, the last, most N-terminal mutant could not (**Figure 3C**), suggesting that the MoHrip2 C-terminus might have a key function inducing HR. To verify this hypothesis, the MoHrip2 C-terminus was expressed (the N-terminus was deleted, MoHrip2Δ N75), and an HR induction assay was performed. The result showed that the C-terminus induced HR (**Figure 3C**), indicating that the above hypothesis was correct.

To further pinpoint the region responsible for HR induction, the middle part of MoHrip2 was isolated, and three truncated mutants, MoHrip2M50 (amino acid sequence from 76 to 125), MoHrip2M36 (amino acid sequence from 89 to 125), MoHrip2M14 (amino acid sequence from 76 to 89) were constructed based on the experimental results (**Figure 3A**). The HR induction assay showed that the MoHrip2M50 and MoHriop2M14 mutants induced HR in tobacco leaves, whereas MoHrip2M36 could not (**Figure 3C**). MoHriop2M14 and MoHrip2M36 were two independent parts of MoHrip2M50 (**Figure 3A**). The amino acid sequence of MoHrip2M14 was located in the central region of the polypeptide chain of MoHrip2 and contained 14 amino acid residues as shown in **Figure 1D**. All of the HR induction assays were performed using full-length MoHrip2 as the positive control and His_6_-Trx as the negative control (**Figure 3C**). To show all of the results in one tobacco leaf, *N. tabacum* cv. Samsun NN was used in the HR induction assay. The experiment was also performed on *N. benthamiana* and showed the same results as *N. tabacum* cv. Samsun NN (**Supplementary Figure S3**).

**FIGURE 4:**
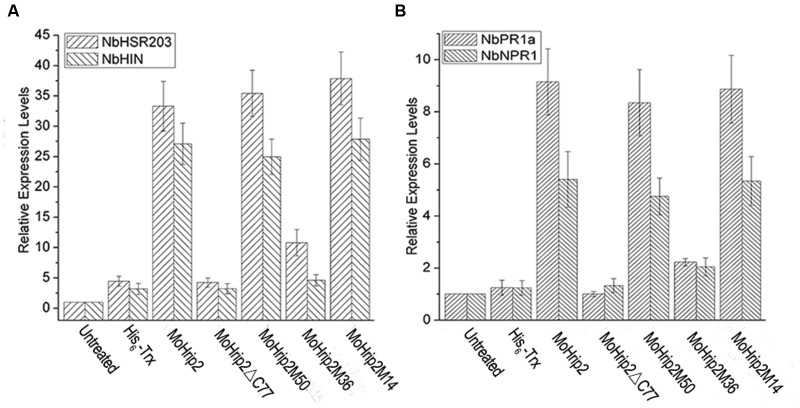
**Quantitative real-time PCR (qRT-PCR) results for representative HR and SAR marker genes in *Nicotiana benthamiana* leaves.** **(A)** Relative expression levels of the HR marker genes *HSR023* and *HIN*1. **(B)** Relative expression of SAR marker genes *NPR*1 and *PR1a*. The relative expression level of each gene was calculated as the fold change 6 hpi in leaves treated with the indicated proteins at a concentration of 5 μM compared to untreated leaves. The *Actin* gene was used as an internal control to normalize the expression data. The data presented are the mean ± standard deviation (SD) of three independent experiments, which showed similar results. The bars represent the SD (*n* = 3).

The *HSR203* and *HIN1* genes are considered HR molecular markers (Liu et al., 2006). The transcriptional level of these two genes was detected using qRT-PCR to verify whether HR induction was coincident with gene expression in the treated leaves. Using the *actin* gene as an internal control, qRT-PCR showed that the infiltration of leaves with MoHrip2, MoHrip2M50 or MoHrip2M14 induced up-regulation of *HSR203* and *HIN1* transcription 6 hpi, whereas leaves infiltrated with MoHrip2Δ C77, MoHrip2M36 or His_6_-Trx showed less increase in transcript expression compared to untreated leaves (**Figure 4A**).

All of the above results indicated that the HR-inducing activity of MoHrip2M14 was equivalent to that of full-length MoHrip2, implying that the 14 amino acid residues play a crucial and independent role inducing HR. In other words, the HR-inducing activity of MoHrip2 is determined by these 14 amino acid residues.

### MoHrip2 and HR-Inducing Truncated Fragments Enhance Resistance to *B. cinerea* in *N. benthamiana*

**FIGURE 5:**
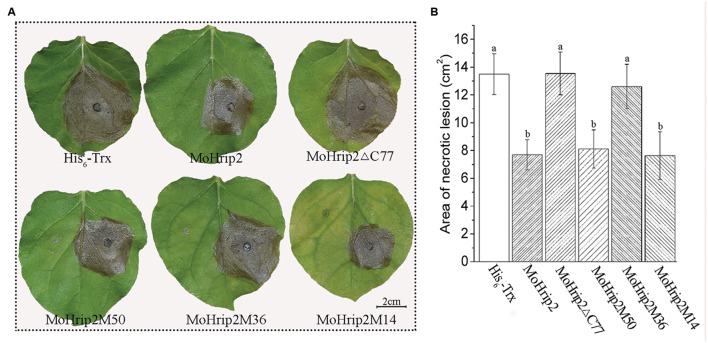
**The effects of the indicated proteins on *Botrytis cinerea* infection in *N. benthamiana*.** **(A)** The symptoms of *B. cinerea* infection 3 dpi. **(B)** Necrotic lesion area caused by *B. cinerea* infection 3 dpi. *B. cinerea* was inoculated 6 hpi on *N. benthamiana* leaves treated with the indicated proteins at a concentration of 5 μM. The data presented were the mean ± SD of three independent experiments that showed similar results. The bars represent the SD (*n* = 3). Different letters on SD bars indicated significant differences between compared plants using one-way ANOVA and Fisher’s Least (LSD) test (*P* < 0.01).

Considering that the MoHrip2M36 and MoHrip2M14 fragments showed significant differences in HR induction ability in *N. benthamiana*, a resistance assay was performed with MoHrip2, MoHrip2Δ C77 and the three middle fragments to test their ability to improve resistance to *B. cinerea.* His_6_-Trx was used as the negative control. *N. benthamiana* leaves were treated with different protein fragments and then incubated with *B. cinerea* mycelium, and the area of necrotic lesions was determined 3 dpi. The necrotic lesions in leaves treated with His_6_-Trx, MoHrip2Δ C77 and MoHrip2M36 were larger than in leaves treated with MoHrip2, MoHrip2M50, or MoHrip2M14 (**Figure 5A**). The area of the lesions was computed by measuring the lesion diameter or the major axes of oval-shaped lesions. The average lesion areas for His_6_-Trx-, MoHrip2Δ C77- and MoHrip2M36-treated leaves were 13.5 ± 1.48 cm^2^, 13.5 ± 1.54 cm^2^, and 12.6 ± 1.60 cm^2^, respectively, whereas, the average lesion areas for MoHrip2-, MoHrip2M50-, and MoHrip2M14-treated leaves were 7.7 ± 1.8 cm^2^, 8.1 ± 1.37 cm^2^, and 7.6 ± 1.72 cm^2^, respectively (**Figure 5B**).

*PR1a* and *NPR1* are important pathogenesis-related genes and systemic acquired resistance (SAR) pathway genes that are regarded as disease resistance marker genes. The transcript levels of the two genes, detected using qRT-PCR, were obviously up-regulated by the infiltration of MoHrip2, MoHrip2M50, or MoHrip2M14 into *N. benthamiana* leaves 6 hpi, but less increase was observed in leaves infiltrated with MoHrip2Δ C77, MoHrip2M36 or His_6_-Trx compared to untreated leaves (**Figure 4B**). The gene *actin* was used as the internal control gene.

These results indicated that MoHrip2 significantly enhanced *N. benthamiana* resistance against the fungal pathogen *B. cinerea* and that the 14 amino acid residues have a crucial role in the induced resistance to pathogens. These results show that the functional region of MoHrip2 responsible for HR induction and disease resistance are in the same short region that contains the 14 crucial amino acid residues.

## Discussion

The MoHrip2 protein is a novel elicitor that was purified in our lab (NCBI locus XP_003709559) from *M. oryzae* 70-15; thus, there is little known about the functional regions of this protein. Our previous study showed that the MoHrip2 amino acid sequence was highly similar to that of proteins from several plant fungal pathogens (Chen et al., 2014). However, no additional information has been reported for these proteins.

In this study, the crystal structure of the protein elicitor MoHrip2 was determined. On the basis of the crystal structure, MoHrip2 showed a high similarity to the three-dimensional structure of a large family of proteins containing a thaumatin-like domain (**Figure 2** and **Table 2**) despite the low homology of their primary sequences (**Supplementary Figure S2**). Of these proteins, we were interested in xylanase inhibitor TL-XI, to which MoHrip2 was most similar in three-dimensional structure (**Figure 2A**). The structure of MoHrip2 is also similar to that of the barrel domain of Beta-1,3-Glucanase (**Figure 2B**), which consists of a barrel domain and a mixed (α/β) domain, with a groove between the two domains. However, the MoHrip2 structure does not contain a mixed domain or the groove that is critical for binding the laminaritetraose (Wu et al., 2009), indicating that some inhibitors might have evolved from protease ancestors (Sansen et al., 2004) and highlighting the value of investigating the evolutionary history of MoHrip2.

The inhibitory effect of TL-XI on xylanase is just one example of an enzyme–inhibitor interaction at the plant–pathogen interface. To invade plant cells, pathogens secrete several enzymes that degrade the cell wall in the plant apoplast, such as endo-β-1,4-xylanases (class GH10 and GH11) that degrade xylan, GH12 glucanases that degrade xyloglucan and polygalacturonases (PGs; class GH28) that hydrolyze homogalacturonan (Juge, 2006). In response, plants secrete many inhibitors to inhibit pathogen enzymes and thus protect themselves from invasion. In addition to TL-XI, several plant inhibitors, such as the TAXI, xylanase inhibitor protein (XIP), and polygalacturonase-inhibiting proteins (PGIPs) have been reported (Misas-Villamil and Ra, 2008). Similarly, plants also secrete many hydrolases or proteinases that degrade the pathogen cell wall, but these molecules are targeted by pathogen-produced inhibitors such as glucanase inhibitor protein-1 (GIP1), which is secreted by *Phytophthora sojae* and targets the specific soybean endo-β-1,3-glucanases (Rose et al., 2002), AVR2 (avirulence protein 2) from the tomato fungal pathogen, and *Cladosporium fulvum*, which targets the tomato papain-like protease (Rooney et al., 2005). Interestingly, some inhibitor–enzyme complexes can act as elicitors and activate the plant defense system, including a complex of the protease RCR3 (required for *Cladosporium* resistance-3) with the inhibitor AVR2, which is recognized by the LRR receptor in tomato (Rooney et al., 2005). We hypothesize that MoHrip2 might be an inhibitor that can act with an enzyme(s) at the plant–pathogen surface when *M. oryzae* infects rice. One possibility is that MoHrip2 inhibits a degradation enzyme from *M. oryzae* to weaken pathogenicity at the stage of invading living rice cells (Qutob et al., 2002; Kankanala et al., 2007). The inhibitory effect of MoHrip2 on different enzymes from pathogens or plants will be tested in future studies to confirm the function of MoHrip2 during pathogenic infection. It is worth noting that all of the thaumatin-domain contained proteins with similar structures to MoHrip2 come from plants, which might be because of that this protein family is mainly found in plant and has not been reported in fungus.

Our results showed that MoHrip2 could enhance resistance to *B. cinerea* in tobacco (**Figure 5**) and induce the expression of pathogenesis-related genes and SAR pathway genes (**Figure 4B**), indicating that the MoHrip2 protein elicitor triggered a defense response to different pathogens in different plants. As an elicitor, the function of MoHrip2 in inducing HR and triggering a defense response to TMV in tobacco and to *M. oryzae* in rice had been reported in a previous study (Chen et al., 2014). As shown, MoHrip2 could induce cell death while enhance resistance to a necrotrophic pathogen, *B. cinerea* (**Figures 3C** and **5**). Both HR marker genes and pahtogenesis-related genes were obviously up-regulated induced by MoHrip2 (**Figure 4**), indicating that MoHrip2 could activate diverse signaling pathways. Furthermore, Several protein elicitors were reported to play functions in different plants against different diseases. For examples, Hrip1, an HR-inducing protein isolated from the fungus *Alternaria tenuissima*, enhances disease resistance to TMV in tobacco and to *B. cinerea* in *Arabidopsis* (Kulye et al., 2012; Peng et al., 2015). The protein elicitor PevD1, which was isolated from the fungal pathogen *Verticillium dahlia*, enhances resistance to TMV in tobacco and to *Verticillium* wilt in cotton (Bu et al., 2014; Liu et al., 2014). All of these results indicate that some protein elicitor showed a broad range of function on disease resistance inducing in different treated plants.

To identify the functional region of MoHrip2 required for its activity, eight truncated mutants were expressed and purified, and the ability of MoHrip2 and the mutants to induce HR and defense responses was investigated. The results indicated that different regions of MoHrip2 had different effects on induction ability. The 14 amino acid residues located in the middle region form the functional region responsible for the HR and the disease-resistance induction (**Figures 3** and **5**) of the MoHrip2 elicitor. These 14 residues include a β-strand within a β-barrel and part of two neighboring loops (**Figure 1D**). During the process of HR induction when interacting with its target, MoHrip2 might undergo local conformational changes around this stretch, especially in the loop regions, to expose active residues necessary for function. Previous reports have indicated that functional region can be located in the same or different regions of an elicitor. The region between amino acids 35 and 53 of HapG, identified in *Xanthomonas axonopodis* pv. *glycines*, induced HR and exhibited elicitor activity (Kim et al., 2004). HpaG_62-137_, a region of HpaG_Xooc_ identified in *Xanthomonas oryzae* pv. *oryzicola*, induced HR in tobacco as well as stimulated a defense response and enhanced growth in rice (Chen et al., 2008). However, although the HpaG_10-42_ region did not induce HR in tobacco, it stimulated a stronger defense response and enhanced more growth in rice than the full-length parental protein (Zhao et al., 2014). The C-terminal 57 amino acid residues of the elicitor PevD1 are critical for inducing HR; however, the N-terminal 98 amino acid residues do not induce HR but retained the ability to enhance resistance against TMV in tobacco (Liu et al., 2014). The 14 amino acid residues in the middle region of MoHrip2 retained both the ability to induce HR and to enhance resistance in tobacco.

## Conclusion

We solved the three-dimensional structure of the elicitor protein MoHrip2, which showed a high similarity to PR-5 family proteins containing a thaumatin-like domain. Mutation and deletion analysis based on the structure of MoHrip2 specified the region that is essential for MoHrip2 elicitor function. A stretch of 14 amino acid residues in the central region of MoHrip2 play essential and independent roles in inducing HR and enhancing disease resistance in tobacco. These results provide a framework for understanding MoHrip2 function at the molecular level in *M. oryzae* and in plants in general. Considering their effects on plant growth and the possibility that overexpression can induce disease resistance (Zhao et al., 2014), short genes might cause fewer adverse effects in transgenic plants. Moreover, as HpaG_10-42_ showed more activity promoting plant growth and defense than HpaG (Chen et al., 2008), the short active region of MoHrip2 might increase the defensive capabilities *in vitro* or in transgenic plants. We will address these issues in future studies.

## Author Contributions

DQ, XL, and ML conceived the project and designed the experiments. ML purified and crystallized MoHrip2, designed and expressed mutant proteins and analyzed mutant protein function in plant. XL determined the X-ray structure. ML, MW, and LD analyzed the data. ML and MW wrote the paper. HZ contributed essential materials. All of the authors reviewed the manuscript.

## Conflict of Interest Statement

The authors declare that the research was conducted in the absence of any commercial or financial relationships that could be construed as a potential conflict of interest.

## Footnotes

1 http://pfam.xfam.org/

2 http://smart.embl-heidelberg.de/
