# Supplementary material for: Crystal Structure Analysis and the Identification of Distinctive Functional Regions of the Protein Elicitor Mohrip2
Source: Front Plant Sci. 2016 Jul 26;7:1103. doi: 10.3389/fpls.2016.01103 (PMC4960229; doi:10.3389/fpls.2016.01103)
Supplement: Supplementary file 1 [file Presentation_1.PDF]

Supplementary Material

Crystal Structure Analysis and the Identification of Distinctive Functional Regions of the Protein Elicitor Mohrip2

Running title: MoHrip2 structure and functional regions

Mengjie Liu[1], Liangwei Duan[2], Meifang Wang[1], Hongmei Zeng[1], Xinqi Liu[2*], Dewen Qiu[1*]

*** Correspondence:** Dewen Qiu: qiudewen@caas.cn; Xinqi Liu: liu2008@nankai.edu.cn

1 Supplementary Figures and Tables

1.1 Supplementary Figures

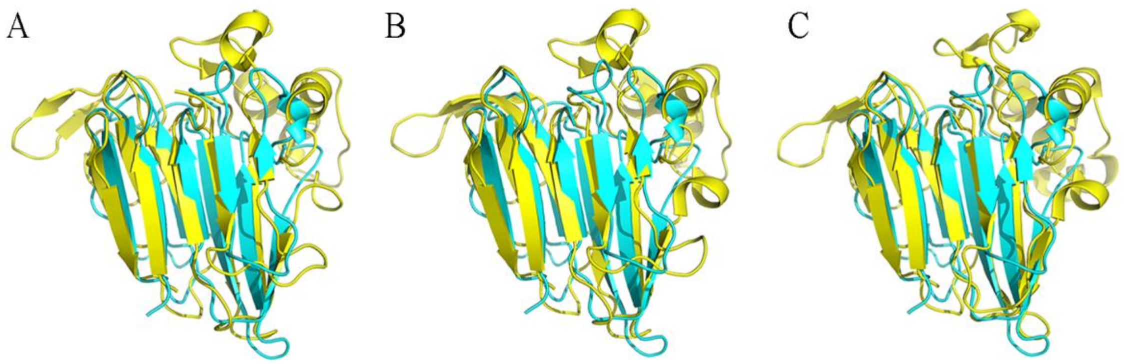

Figure S1. The comparison of protein crystal structures. **(A)** Superimposed structures of MoHrip2 and NP24 (PDB 2IOW). **(B)** Superimposed structures of MoHrip2 and osmotin (PDB 1PCV). **(C)** Superimposed structures of MoHrip2 and zeamatin (PDB 1DU5). All of the structures are shown in ribbon representation. MoHrip2 is colored cyan, and the others are colored yellow.

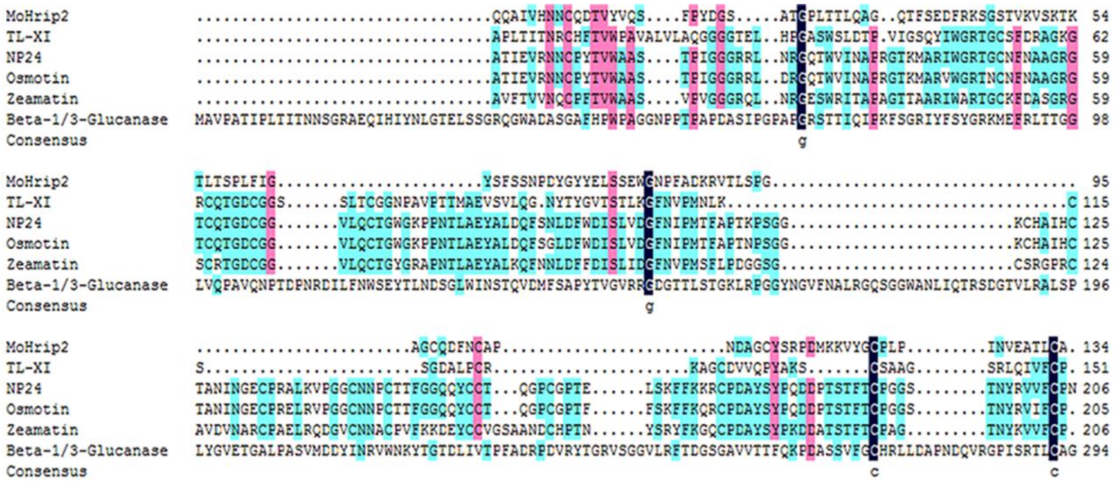

Figure S2. Amino acid sequence alignment of MoHrip2 with several representatives of the thaumatin protein family. TL-XI (gi|291191097), NP24 (gi|157835146), osmotin (gi|46015197), zeamatin (gi|6980846) and Beta-1,3-Glucanase (gi|254839318) from *Triticum aestivum*, *Solanum lycopersicum*, *Nicotiana tabacum*, *Zea mays*, and the actinobacteria *Streptomyces matensis*, respectively. Identical residues are colored midnight blue, 75% similar residues are magenta, and 50% similar residues are cyan.

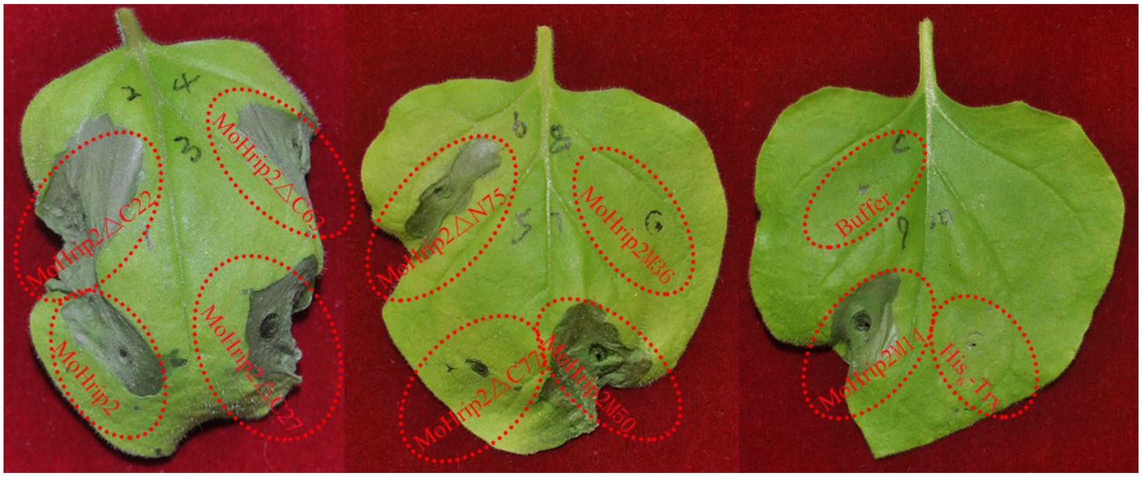

Figure S3. Macroscopic views of HR symptoms induced by the prepared proteins at a concentration of 5 mM in *Nicotiana tabacum*. The photos were taken 24 h after infiltration.

1.2 Supplementary Tables

Table S1 Primer sequences used in this study.
The underlined letters are vector sequence.

Product	Primers sequence	Description
MoHrip2	TACTTCCAATCCAATGCCCAGCAGGCTATCGTCCACAAC	pET-30 TEV/LIC vector
	TTATCCACTTCCAATGCTATTAGGCGCAGAGGGTAGCCTC	
MoHrip2△ C22	TACTTCCAATCCAATGCCCAGCAGGCTATCGTCCACAAC	
	TTATCCACTTCCAATGCTATTAGCTGTAGCAGCCGGCGTC	
MoHrip2△ C27	TACTTCCAATCCAATGCCCAGCAGGCTATCGTCCACAAC	
	TTATCCACTTCCAATGCTATTAGTCGTTGGGGGCGCAGTTG	
MoHrip2△ C63	TACTTCCAATCCAATGCCCAGCAGGCTATCGTCCACAAC	
	TTATCCACTTCCAATGCTATTAGTCCGGGTTCGACGAGAACC	
MoHrip2△ C77	CGGGATCCCAGCAGGCTATCGTCCACAAC	pET-M3C vector
	CCGCTCGAGTTAGCTGGTCAAGGTCTTGGTC	
MoHrip2△ N75	CGGGATCCAGCCCTCTGTTCATCGGCTAC	
	CCGCTCGAGTTAGGCGCAGAGGGTAGCCTC	
MoHrip2M50	CGGGATCCAGCCCTCTGTTCATCGGCTAC	
	CCGCTCGAGTTAGTCGTTGGGGGCGCAGTTG	
MoHrip2M36	CGGGATCCTACGGCTACTATGAGCTGTCC	
	CCGCTCGAGTTAGTCGTTGGGGGCGCAGTTG	
MoHrip2M14	CGGGATCCAGCCCTCTGTTCATCGGCTAC	
	CCGCTCGAGTTAGTCCGGGTTCGACGAGAAC	
Actin (X69885)	GGTCGTACAACTGGTATTG	quantitative RT-PCR
	GAGGATCTTCATCAGGTTATC	
HSR203 (AF212184)	CTATCATTGTCTCCGTCTTC	
	ATCTGCGTAATCGTTGAG	
HIN (Y07563.1)	CTTGCGTCCAGTATTCAA	
	GCTTCACTTCCATCTCATAA	
PR1a (D90196)	GGTTCAGTGTAACAATGGA	
	TATGGACTTTCGCCTCTATA	
NPR1 (AF480488)	GGAATGATACGGCAGAAG	
	AGGAACAAGATGAGGAGAT	

Table S2 The detailed characteristics of purified proteins used in this study.

Purified Protein	pI[a]	AA No.[b]	M.W.[c]	Vector used	Tag(s)	M.W. (with tag)[d]	solubility
MoHrip2	5.10	134	14.53	pET-30 TEV/LIC	His_6	17.27	inclusion bodies
MoHrip2△ C22	4.64	112	12.16			15.03	
MoHrip2△ C27	4.64	107	11.65			14.52	
MoHrip2△ C63	7.27	71	7.70			10.56	
MoHrip2△ C77	8.19	57	6.28	pET-M3C	His_6-Trx	20.77	soluble
MoHrip2△ N75	4.28	77	8.27			22.85	
MoHrip2M50	3.73	50	5.49			19.97	
MoHrip2M36	3.89	36	3.97			18.46	
MoHrip2M14	3.45	14	1.53			16.02	
His_6-Trx	6.12					17.18	

[a], the theoretical value of isoionic point of indicated fragments.
[b], the number of amino acid residues of indicated fragments.
[c], molecular weight of indicated fragments without tag (kDa).
[d], molecular weight of indicated fragments with tag(s) (kDa).
